# Imaging Effector Memory T-Cells Predicts Response to PD1-Chemotherapy Combinations in Colon Cancer

**DOI:** 10.3390/biomedicines10102343

**Published:** 2022-09-20

**Authors:** Julian L. Goggi, Shivashankar Khanapur, Siddesh V. Hartimath, Boominathan Ramasamy, Peter Cheng, Hui-Xian Chin, Jun-Rong Tang, You-Yi Hwang, Edward G. Robins

**Affiliations:** 1 Institute of Bioengineering and Bioimaging (IBB), Agency for Science, Technology and Research (A*STAR), 11 Biopolis Way, #01-02 Helios, Singapore 138667, Singapore; 2 Singapore Immunology Network (SIgN), Agency for Science, Technology and Research (A*STAR), 8A Biomedical Grove, Immunos, Singapore 138648, Singapore; 3 Clinical Imaging Research Centre (CIRC), Yong Loo Lin School of Medicine, National University of Singapore, 14 Medical Drive, #B1-01, Singapore 117599, Singapore

**Keywords:** effector memory T-cell, Kv1.3 potassium channel, chemotherapy, immune checkpoint

## Abstract

Often, patients fail to respond to immune checkpoint inhibitor (ICI) treatment despite favourable biomarker status. Numerous chemotherapeutic agents have been shown to promote tumour immunogenicity when used in conjunction with ICIs; however, little is known about whether such combination therapies lead to a lasting immune response. Given the potential toxicity of ICI–chemotherapy combinations, identification of biomarkers that accurately predict how individuals respond to specific treatment combinations and whether these responses will be long lasting is of paramount importance. In this study, we explored [^18^F]AlF-NOTA-KCNA3P, a peptide radiopharmaceutical that targets the Kv1.3 potassium channel overexpressed on T-effector memory (T_EM_) cells as a PET imaging biomarker for lasting immunological memory response. The first-line colon cancer chemotherapies oxaliplatin and 5-fluorouracil were assessed in a syngeneic colon cancer model, either as monotherapies or in combination with PD1, comparing radiopharmaceutical uptake to memory-associated immune cells in the tumour. [^18^F]AlF-NOTA-KCNA3P reliably separated tumours with immunological memory responses from non-responding tumours and could be used to measure Kv1.3-expressing T_EM_ cells responsible for durable immunological memory response to combination therapy in vivo.

## 1. Introduction

Immune checkpoint inhibitors (ICIs) have transformed the field of immunotherapy; however, many patients do not respond to ICI monotherapy, despite favourable biomarker status (PD-L1, tumour mutational burden or microsatellite instability) [1,2,3]. The efficacy of immune checkpoint inhibitors (ICIs) is influenced by alterations in the tumour microenvironment, where suppression and resistance mechanisms, such as mutations in key effector pathways or in immune effector signalling pathways, can lead to low durable response rates [4,5]. In an effort to enhance response rates to ICIs, many studies have tried combining different ICIs together, such as αPD1 and αCTLA4. Unfortunately, ICI combinations can cause severe immune-related side effects. CTLA4 is widely distributed and blockade leads to diverse immune-related side effects including colitis and hepatitis [6,7]. PD1 is mainly limited to immune cells, so side effects caused by PD1 blockade tend to be less severe. Thus, clinical trials evaluating therapeutic combinations mainly focus on enhancing responsiveness to αPD1 by ameliorating tumour immunogenicity. Numerous anti-cancer drugs have been shown to promote tumour immunogenicity through mechanisms such as immunogenic cell death and modulation of tumour cell surface regulators [8]. Both 5-fluorouracil (5-FU) and oxaliplatin (OXA), chemotherapeutics used in the first-line treatment of colorectal cancer, increase tumour immunogenicity when used in conjunction with PD1 [9]. OXA induces immunogenic cell death, releasing damage-associated molecular patterns (DAMPs) [10] and resulting in an increase in tumour-infiltrating CD8^+^ T-cells [9,11,12,13], whereas 5-FU both reduces tumour-associated immune-suppressive cells and increases tumour-infiltrating NK cells [9,14,15]. They improve treatment response when combined with αPD1 [9,16]. However, little is known about whether combinations of these chemotherapeutics with ICIs lead to a durable response that is likely to improve overall survival long-term. The high cost and potential toxicity associated with ICI–chemotherapeutic combinations necessitate the identification of biological markers that accurately predict how individuals respond to specific treatment combinations and whether these responses will be long lasting; however, such assessments are complex. Previous studies have demonstrated that immunological memory responses are required for durable response to ICI therapy [17,18,19]; in particular, tumour infiltration of active effector memory T-cells (T_EM_ cells) [20]. T_EM_ cells display superior antitumor efficacy. as they have lower activation thresholds than naïve T-cells (responding to 100-fold lower doses of antigen) and respond more rapidly to stimulation [21]. Furthermore, T_EM_ cells have an enhanced capacity to migrate to lymph nodes and areas of inflammation. Overall, the presence of high levels of infiltrating T_EM_ cells correlates well with the absence of metastatic invasion and increased survival [22]. Activated T_EM_ cells (CD45RO^+^CCR7^−^) express high levels of K_V_1.3 potassium channels, while K_Ca_3.1 channels are more abundant in activated naïve (CD45RO^−^CCR7^+^) and T_CM_ cells (CD45RO^+^CCR7^+^) [23,24,25]; hence, the expression levels of Kv1.3 may be used as a biomarker to identify the presence of T_EM_ cells in an effort to stratify durable ICI response. In the current study, we evaluated whether [^18^F]AlF-NOTA-KCNA3P, a peptide probe targeting Kv1.3, is able to reliably stratify lasting therapy response in ICI–chemotherapy treatment combinations. We used flow cytometry to determine which tumour-infiltrating immune cell populations were associated with response to these treatment combinations in a murine syngeneic colorectal cancer model.

## 2. Materials and Methods

### 2.1. [^18^F]AlF-NOTA-KCNA3P Radiochemistry

The precursor NOTA-KCNA3P peptide was custom synthesized by Chinese Peptide Company (CPC) and radiolabelling was performed as previously described [26]. [^18^F]AlF-NOTA-KCNA3P was isolated with a non-decay-corrected radiochemical yield of 12 ± 6 % within 50 min (*n* = 6) from aqueous [^18^F]fluoride. The radiochemical purity was greater than 99% and molar activity was 59 ± 16 GBq/µmol at the end of synthesis (*n* = 6).

### 2.2. Tumour Implantation and Dosing Regimen

**Figure 1:**
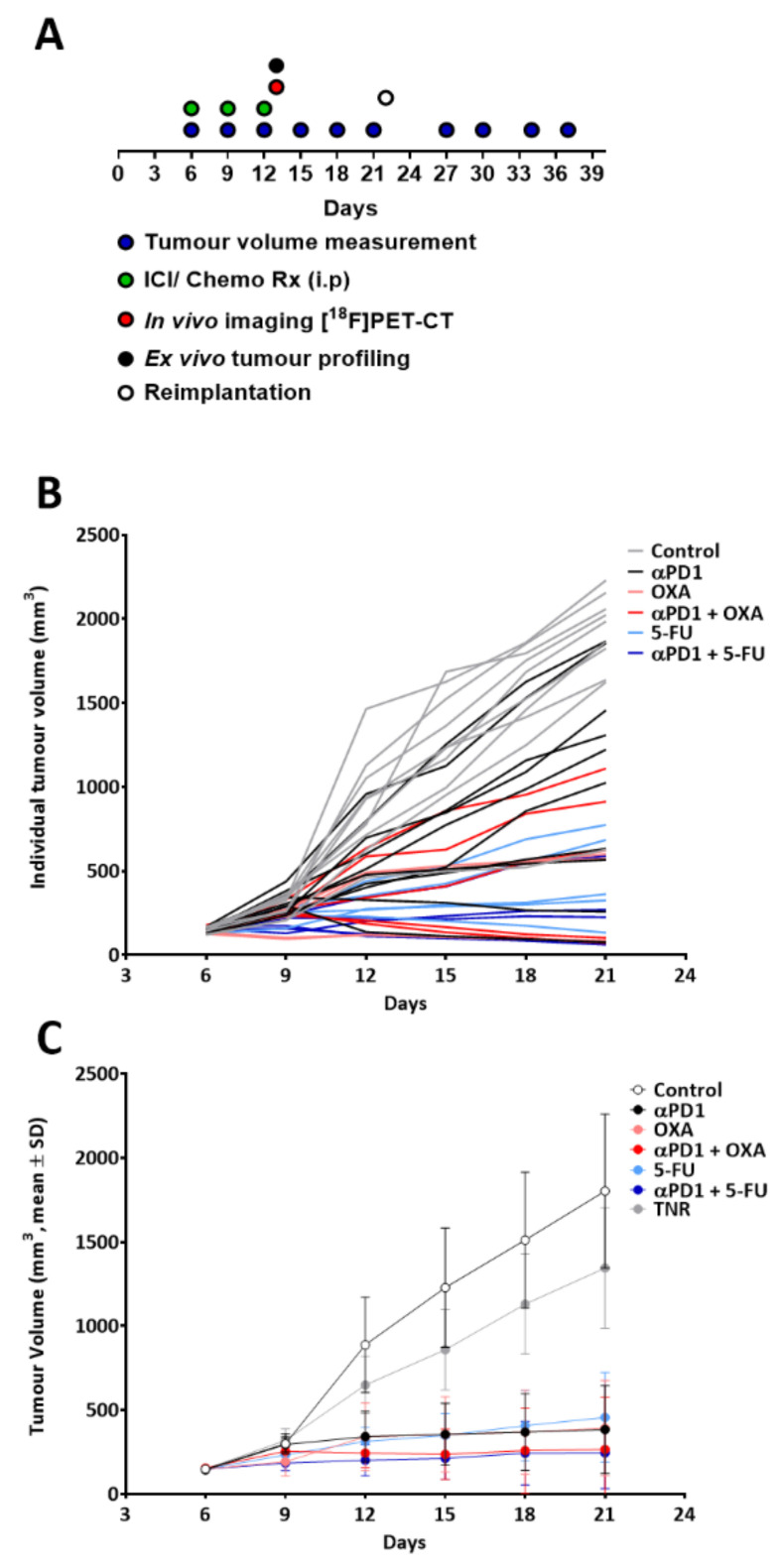
(**A**) Schema representing the dosing, measurement and imaging procedure. Mice (*n* = 10 per treatment arm) were treated with control IgG, αPD1, oxaliplatin (OXA), combination αPD1 + OXA, 5-fluorouracil (5-FU) or combination αPD1 + 5-FU post-tumour implantation. (**B**) Individual animal tumour volumes showing heterogeneity in tumour response. (**C**) Average tumour volume in each treatment arm, post-therapy response stratification. Data are displayed as means ± S.D. TNR, treated non-responder.

All animal procedures adhered to the Singapore Institutional Animal Care and Use Committee regulations (IACUC No. 211649). The CT26 tumour implantation and dosing regimen have been reported previously [9]. Briefly, mice (BALB/c, 5–7 weeks) were purchased from InVivos (Singapore) and injected subcutaneously with CT26 cells (2 × 10^5^ cells prepared in a 1:1 *v*:*v* ratio in Matrigel, Sigma-Aldrich, Singapore) into the right shoulder. Callipers were used to assess tumour volumes for the duration of the experiment (modified ellipsoid formula 1/2Length × Width^2^ [27]). Tumoured animals were treated with either a control (rat IgG2a isotype control, 5 mg/kg, IP (⍺-trinitrophenol mAb, *n* = 10)) or the immune checkpoint inhibitor ⍺PD1 (rat IgG2a anti-mouse PD-1, 10 mg/kg, IP (⍺PD1 mAb RMP1-14, Bio-X-Cell, New Hampshire, USA, *n* = 15)) on days 6, 9 and 12 following tumour implantation. The chemotherapy groups were treated with oxaliplatin (OXA, 6.0 mg/kg, Sigma-Aldrich, Singapore, IP, Q7D, *n* = 10) or 5-fluorouracil (5-FU, 70 mg/kg, IP, Q3D, Sigma-Aldrich, Singapore, *n* = 10) alone or in combination with αPD1 (*n* = 10 per group). Figure 1A shows a schematic of the dosing and assessment regimen. Tumour response to therapy was determined by measuring tumour growth inhibition (%TGI, (V_c_ − V_t_)/(V_c_ − V_o_) × 100, where V_c_ and V_t_ are the mean tumour volumes of control and treated groups on day 21 and V_o_ is the tumour volume at the start of the study) (Supplementary Table S3).

After the animals had been separated into treatment responders (TR) and treatment non-responders (TNRs) based on their response to therapy, TR animals with high [^18^F]AlF-NOTA-KCNA3P tumour retention (>0.8%ID/g) and TNR animals with low [^18^F]AlF-NOTA-KCNA3P tumour retention (<0.5%ID/g) were re-challenged, implanting CT26 tumour cells (2 × 10^5^ per animal) into the contralateral left shoulder. Contralateral tumour re-growth was measured using callipers for a further 15 days.

### 2.3. PET-CT Imaging

Tumoured animals underwent imaging using a Siemens Inveon PET-CT 13 days after tumour implantation as previously described [26]. Briefly, animals were injected with [^18^F]AlF-NOTA-KCNA3P (~10 MBq) via the lateral tail vein, static PET acquisitions were acquired and analysis of reconstructed calibrated images was performed using Amide software (version 10.3 Sourceforge, Stanford, CA, USA). Volumes of interest, delineated by CT imaging, were used to determine uptake in tissues. Data are expressed as percentages of the injected dose per gram (%ID/g).

### 2.4. Flow Cytometry and Dimension Reduction Analysis

The flow cytometry procedure has been reported previously [9]. Briefly, the tumours were excised after in vivo PET imaging and freshly processed for flow cytometry. A single-cell suspension was generated and assessed for viability with Trypan Blue (Sigma-Aldrich, Singapore) with staining for the following markers: Kv1.3, CD103, CD25, CD45, CD62L, CD86, F4/80, NKp46, CD3e, FoxP3, CD44, CD11b, Granzyme B, CCR7, CD19, CD206, CD127, Ly6G, CD8, CD11c, Ly6C, Siglec F, CD68, CD4 and I-A/I-E (clone and manufacturer details in Supplementary Materials Section S1.1). Flow cytometry was performed on a BD FACSymphony, Oregon USA. Data were recompensated and analysed using FlowJo V10.7.1 software (FlowJo LLC, Oregon USA).

Time-gated, size-gated, live, singlet CD45-positive cells from the fcs files were exported from FlowJo and used for dimension reduction analysis as reported previously [9] (for further details, see Supplementary Materials Section S1.2).

### 2.5. Statistical Analysis

Kruskal Wallis one-way ANOVA was used to assess the non-parametric datasets, with a Dunn’s post-test for multiple comparisons. All statistics were evaluated using GraphPad Prism version 8.3.4 (GraphPad Software, San Diego, CA, USA, www.graphpad.com, accessed from 1 January 2022). *p* < 0.05 was considered statistically significant. Data are expressed as means ± S.D. unless otherwise indicated.

## 3. Results

### 3.1. Evaluation of Treatment Efficacy Using Tumour Volumes

Tumour volumes were normally distributed prior to treatment separation (Shapiro–Wilk *p* = 0.6378). Treatment arms including oxaliplatin, 5-FU and αPD1 were administered on their own or in pairwise ICI–chemotherapy combinations (Figure 1). Each treatment arm showed varying rates of response and varying extent of tumour growth inhibition depending on the therapeutic intervention studied. Figure 1B shows the individual animal tumour volumes for each treatment arm. The stratification of tumours responding to treatment (TR) and those not responding (TNRs, Figure 1C) has been described previously [9]. TRs were defined by day 21 tumour volumes less than 880 mm^3^ (<2 SD mean volume of the control group on day 21, when the control group reached the size limit on out license). Combined ICI–chemotherapy treatment arms had both greater response rates and response magnitudes than αPD1 or chemotherapy monotherapy arms (Supplementary Tables S2 and S3).

### 3.2. In Vivo PET Imaging with [^18^F]AlF-NOTA-KCNA3P

**Figure 2:**
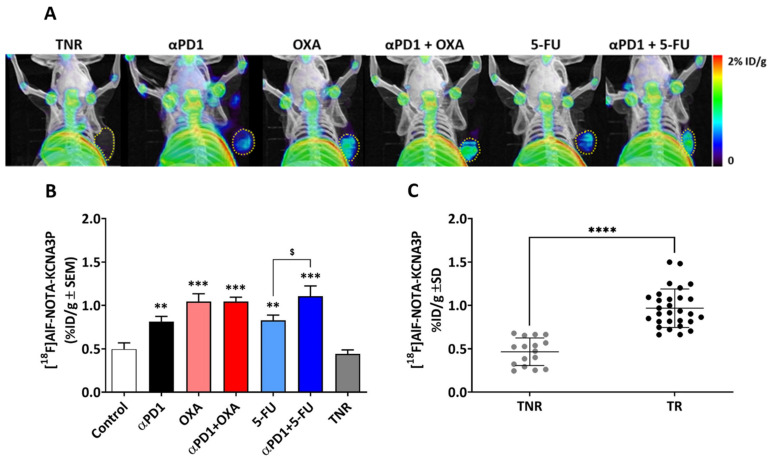
(**A**) Selected MIP images demonstrating [^18^F]AlF-NOTA-KCNA3P uptake into tumours responding to αPD1, oxaliplatin (OXA), combined ⍺PD1 + OXA, 5-fluorouracil (5-FU), combined αPD1 + 5-FU or non-responding tumours (TNRs). Tumour borders are delineated by yellow dotted lines. (**B**) Graphical representation of [^18^F]AlF-NOTA-KCNA3P tumour uptake values in each treatment arm (*n* = 6–10 mice/group; ** *p* < 0.01, *** *p* < 0.001 compared to TNRs; ^$^ *p* < 0.05 compared to 5-FU; data shown as the means %ID/g ± S.E.M.). (**C**) Individual [^18^F]AlF-NOTA-KCNA3P tumour values comparing TRs and TNRs (*n* = 46, **** *p* < 0.0001).

[^18^F]AlF-NOTA-KCNA3P showed good tumour uptake and background was low. Tumour uptake was mixed across the animals studied, depending on treatment exposure and response (Figure 2A). Overall, [^18^F]AlF-NOTA-KCNA3P tumour uptake and tumour growth inhibition were correlated across all the animals studied (Pearson r = 0.831, **** *p* < 0.0001, *n* = 60). The control-treated arm and TNRs showed little tumour retention for [^18^F]AlF-NOTA-KCNA3P. The ⍺PD1- and 5-FU-responsive tumours both showed significant increases compared to TNRs (** *p* < 0.01 for both). The OXA responders (*** *p* < 0.001) and the ⍺PD1 + OXA and ⍺PD1 + 5-FU responders (*** *p* < 0.001 for both) showed significantly higher tumour uptake of [^18^F]AlF-NOTA-KCNA3P when compared to TNRs (Figure 2B and Table 1).

To further assess whether [^18^F]AlF-NOTA-KCNA3P tumour uptake predicted durable, lasting treatment efficacy, the animals were re-implanted with tumours (CT26 cells) in the contralateral shoulder and tumour growth was measured for 15 days after implantation. TR animals with high tumour uptake of [^18^F]AlF-NOTA-KCNA3P on day 13 (>0.8%ID/g) showed negligible tumour regrowth after re-challenge up to 15 days post-re-implantation in the contralateral shoulder, whereas TNR animals with low tumour uptake of [^18^F]AlF-NOTA-KCNA3P showed significant tumour growth after re-implantation (Supplementary Figure S1 and Supplementary Table S5). These results highlight the effectiveness of the [^18^F]AlF-NOTA-KCNA3P tumour-uptake imaging assay in predicting durable treatment efficacy.

### 3.3. Tumour Infiltration of Effector Memory T-Cells Is Responsible for [^18^F]AlF-NOTA-KCNA3P Tumour Uptake

**Figure 3:**
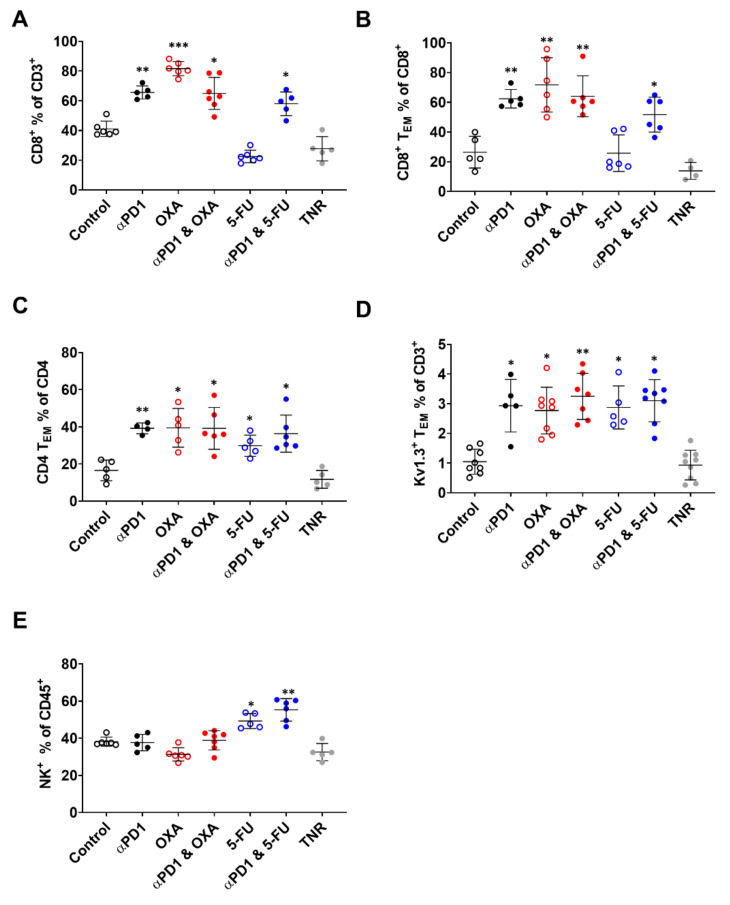
Flow cytometry depicting tumour-infiltrating **i**mmune cell populations in each treatment arm. Percentages of (**A**) CD8^+^ T-cells relative to CD3^+^ cells, (**B**) CD8^+^ T_EM_ cells relative to total CD8^+^ cells, (**C**) CD4^+^ T_EM_ cells relative to total CD4^+^ cells, (**D**) Kv1.3^+^ T_EM_ cells relative to total CD3^+^ cells and (**E**) NK^+^ cells relative to total CD45^+^ cells across all treatment arms. Data are individual values with the mean ± S.D. and are representative of *n* = 4–7 mice/group. * *p* < 0.05; ** *p* < 0.01; *** *p* < 0.001 compared to TNR.

Flow cytometry was used to assess which immune cell populations were associated with tumours that responded to therapy (TR) compared to tumours that did not respond to therapy (TNR; Figure 3, Table 2 and Supplementary Table S4). Earlier studies have shown that OXA treatment increases tumour infiltrating CD8^+^ T-cells [9,11,12,13] whereas 5-FU increases tumour-infiltrating NK cells [9,14,15]. Here, assessment of immunophenotypic changes across the different treatment arms clearly showed that, in tumours responding to ⍺PD1 or OXA (when administered as a monotherapy or in combination), the most significant differences in immune cells were observed for tumour-infiltrating CD8^+^ T-cells, tumour infiltrating CD8^+^ T_EM_ cells and CD4^+^ T_EM_ cells (Figure 3A–E, Table 2). In contrast, tumours responding to 5-FU (when administered as monotherapy or in combination) showed significant increases in tumour-associated NK^+^ cells and CD4^+^ T_EM_ cells (Figure 3C,E, Table 2).

## 4. Discussion

The immune checkpoint PD-1 receptor is predominantly responsible for the regulation of T-cell responses. Unfortunately, blockade of PD-1 not only activates tumour-associated T-cells but also triggers activation of compensatory T-cell-associated checkpoints, which can limit the duration of ICI efficacy [2,4,5,28]. Adjuvant therapies that enhance the immune environment have been an area of intense research in a bid to bolster αPD1 efficacy and duration of response [29,30,31]. Imaging with [^18^F]AlF-NOTA-KCNA3P has previously been shown to reliably stratify tumours responding to immune checkpoint inhibitors from non-responding tumours, measuring tumour-associated Kv1.3-expressing T_EM_ cells responsible for durable immunological memory response to combination therapy in vivo; however, little is known about whether adjuvant therapies that modulate the immune environment will synergise with the effects of PD1 blockade to promote a lasting immunological memory response at the tumour. Immunological memory occurs when naïve T-cells are repeatedly exposed to antigens and differentiate into memory T-cells, antigen-specific T-cells that remain long-term and rapidly proliferate in response to antigen re-exposure. Effector memory T-cells (T_EM_), in particular, are associated with durable tumour response to ICIs [19,20,32]. Treatments with αPD1 and combined αPD1 + αCTLA4 have previously been shown to substantially increase tumour-infiltrating CD4^+^ and CD8^+^ T_EM_ cells [26].

OXA and 5-FU are chemotherapeutics used in the first-line treatment of colon cancer and both have previously been observed to profoundly affect the immune system, improving response when combined with αPD1 [9,16,33]. OXA induces immunogenic cell death, releasing damage-associated molecular patterns (DAMPs) [10] and resulting in an increase in tumour-infiltrating T-cells [9,11,12,13], whereas 5-FU both reduces tumour-associated immune-suppressive cells and increases tumour-infiltrating NK cells [9,14,15]. In the current study, repeated dosing with the platinum-based chemotherapeutic OXA likewise significantly increased tumour-associated CD8^+^ and CD4^+^ T_EM_ cells (*p* < 0.01 and *p* < 0.05 respectively); however, while the effect was still significant when dosed in combination with αPD1 (*p* < 0.01), there was no evidence of synergy, suggesting that OXA alone may maximally recruit T_EM_ cells. These increases in tumour-associated T_EM_ cells were mirrored by significant increases in Kv1.3-expressing T_EM_ cells and tumour retention of [^18^F]AlF-NOTA-KCNA3P (Table 1, Figure 3B–D), showing a clear correlation between tumour-infiltrating T_EM_ cells and radiopharmaceutical uptake.

5-FU treatment led to increases in tumour-infiltrating NK cells as previously reported [9], but no increases in CD8^+^ memory cells were observed (Figure 3B). Furthermore, when 5-FU was dosed in combination with αPD1, increases in tumour-associated CD8^+^ T_EM_ cells were equivalent to changes observed after αPD1 monotherapy alone. However, tumour uptake of [^18^F]AlF-NOTA-KCNA3P was increased in tumours responding to 5-FU and αPD1 + 5-FU, despite the apparent lack of CD8^+^ T_EM_ cell response. Further interrogation of the FACS data showed that the tumour-infiltrating NK cells associated with 5-FU treatment did not express high levels of Kv1.3; hence, [^18^F]AlF-NOTA-KCNA3P was instead likely measuring increases in tumour-infiltrating Kv1.3-expressing CD4^+^ T_EM_ cells (Figure 3D). Unlike CD8^+^ T_EM_ cells, which are directly involved in mediating tumour apoptosis, CD4^+^ T_EM_ cells play a supporting role, rapidly producing a cytokine response and reducing CD8^+^ T-cell exhaustion [34]. Despite the difference in immune cell infiltrates after OXA or 5-FU treatment, both were capable of enhancing response to αPD1 therapy and [^18^F]AlF-NOTA-KCNA3P was still able to assess therapy response when the chemotherapeutics were utilised alone or in combination with αPD1. In each responding treatment arm, imaging with [^18^F]AlF-NOTA-KCNA3P precisely measured T_EM_ cell tumour infiltration, providing a non-invasive measure for durable, long-lasting ICI therapy response, exemplified by the lack of tumour regrowth after re-challenge, as shown in Supplementary Figure S1. Whether [^18^F]AlF-NOTA-KCNA3P is able to stratify chemotherapy–ICI combinations and their effect on T_EM_ cell infiltration in the clinic remains to be seen; the immunomodulatory effects of chemotherapeutics are often observed at doses lower than those used for tumour treatment. However, data from the recent KEYNOTE clinical trials suggest that adjuvant chemotherapy dosing improves ICI response (without significant worsening of immune-related adverse events) [35,36], and previous clinical studies have shown that chemotherapy treatment enhances tumour-associated memory T-cells [37]. Overall, the data suggest that [^18^F]AlF-NOTA-KCNA3P could be a useful addition to [^18^F]FDG imaging in the clinic, aiding in therapy management and helping distinguish tumour response from pseudoprogression or immune-related adverse events.

## 5. Conclusions

Although the chemotherapeutic agents assessed in this study affected the tumour microenvironment in different ways, both led to effective immunological memory responses in tumours and complemented the CD8^+^ T_EM_ tumour infiltration induced by PD1 blockade. Imaging with [^18^F]AlF-NOTA-KCNA3P accurately measured Kv1.3-expressing T_EM_ cells associated with durable response to combination therapy in vivo. With further development, [^18^F]AlF-NOTA-KCNA3P may serve as a clinical biomarker to support investigations into new therapy combination strategies to enhance responsiveness to ICIs.

## Figures and Tables

**Table 1:** Table displaying [^18^F]AlF-NOTA-KCNA3P tumour uptake in each treatment arm. Data are represented as %ID/g ± S.D. comparing treated responders from each treatment arm (TR) to treated non-responders (TNRs) (*n* = 6–10 mice/group; ** *p* < 0.01; *** *p* < 0.001 comparing TRs in each treatment arm to TNRs; ^$^ *p* < 0.05 comparing ⍺PD1 + 5-FU TRs to 5-FU TRs).

Treatment Arm	[^18^F]AlF-NOTA-KCNA3P (%ID/g ± SD)
Control	0.50 ± 0.19
Treated Responders (TRs) αPD1	0.81 ± 0.17 **
OXA	1.05 ± 0.18 ***
αPD1 + OXA	1.05 ± 0.11 ***
5-FU	0.83 ± 0.15 **
αPD1 + 5-FU	1.11 ± 0.31 ***^,$^
Treated Non-Responders (TNRs)	0.44 ± 0.14

**Table 2:** Tumour-infiltrating **i**mmune cell populations in each treatment arm. Data are represented as % immune cell population ± S.D. comparing treated responders from each treatment arm (TR) to treated non-responders (TNRs) (*n* = 5–10 mice/group; * *p* < 0.05, ** *p* < 0.01, *** *p* < 0.001 comparing TRs in each treatment arm to TNRs).

	Tumour-Infiltrating Immune Cell Populations				
Treatment Arm	CD8^+^% of CD3^+^	CD8^+^ T_EM_% of CD8^+^	CD4^+^ T_EM_% of CD4^+^	KV1.3^+^ T_EM_% of CD3^+^	NK^+^% of CD45^+^
Control	41.12 ± 5.17	26.43 ± 10.69	16.54 ± 5.57	0.92 ± 0.40	38.16 ± 2.46
TR αPD1	65.63 ± 4.36 **	62.42 ± 6.26 **	39.22 ± 2.91 **	2.63 ± 0.60 *	37.70 ± 4.42
OXA	81.63 ± 4.75 ***	71.70 ± 18.32 **	39.48 ± 10.44 *	3.41 ± 0.72 *	31.33 ± 3.60
αPD1 + OXA	64.96 ± 10.79 *	64.06 ± 13.78 **	39.18 ± 11.26 *	3.06 ± 0.83 **	38.89 ± 5.18
5-FU	22.52 ± 4.21	25.76 ± 12.31	29.83 ± 5.72	2.67 ± 0.76 **	49.28 ± 4.05 *
αPD1 + 5-FU	57.99 ± 7.96 *	51.72 ± 11.73 *	33.16 ± 5.08	3.49 ± 0.82 *	55.31 ± 6.07 **
TNR	27.70 ± 8.18	13.85 ± 5.70	11.73 ± 4.78	0.83 ± 0.45	32.54 ± 4.65

## Data Availability

The data presented in this study are available on request from the corresponding author.

## Supplementary Materials

The following supporting information can be downloaded at https://www.mdpi.com/article/10.3390/biomedicines10102343/s1. **Supplementary Table S1.** Summary of tumour volumes in controls, ICI treatment responders (TR) and treatment non-responders (TNR). **Supplementary Table S2.** Summary of ICI treatment responders (TR) and treatment non-responders (TNR) across all therapy arms in syngeneic CT26 and MC38 colon cancer models. **Supplementary Table S3.** Tumour growth inhibition % on day 21 for each treatment arm compared to control. **Supplementary Table S4.** Table showing the tumour associated immune cell populations from CT26 tumour-bearing mice at day 12 post-induction of αPD1 monotherapy or combination therapies. A. Percentages of CD4^+^, CD4^+^ T-effector, CD4^+^ T-central memory and CD4^+^ T-regulatory immune cell subpopulations and B. Percentages of CD8^+^ T-effector, CD8^+^ T-central memory, F4/80^+^ and Eos^+^ immune cell subpopulations are shown across control groups, treatment responders (TR) and treatment non-responders (TNR) across all treatment arms. Data are shown as mean % of cells ± S.D. and are representative of *n* = 5–10 mice/group, * *p* < 0.05; ** *p* < 0.01 comparing TR to TNR. **Supplementary Table S5.** Summary of tumour volumes in reimplanted TNRs and reimplanted TRs. Data are displayed as mean ± S.D. (TR, treated responder; TNR, treated non-responder) and are representative of *n* = 4–6 mice/group, * *p* < 0.05 comparing TR to TNR. **Supplementary Figure S1.** Average tumour volumes showing initial tumour growth (TR, red closed circles and TNR, closed black circles) and after CT26 tumour cell re-implantation subcutaneously into the contralateral shoulder of TRs (red open circles; animals with high [^18^F]AlF-NOTA-KCNA3P uptake >0.8%ID/g) or TNRs (black open circles; animals with low [^18^F]AlF-NOTA-KCNA3P uptake <0.5%ID/g). Data are displayed as mean ± S.E.M. (TR, treated responder; TNR, treated non-responder).
