# Supplementary material for: Imaging Effector Memory T-Cells Predicts Response to PD1-Chemotherapy Combinations in Colon Cancer
Source: Biomedicines. 2022 Sep 20;10(10):2343. doi: 10.3390/biomedicines10102343 (PMC9598730; doi:10.3390/biomedicines10102343)
Supplement: Supplementary file 1 [file biomedicines-10-02343-s001.zip › biomedicines-1896840-supplementary.pdf]

biomedicines

Supplementary

Imaging effector memory T-cells predicts response to PD1-chemotherapy combinations in colon cancer.

Julian L. Goggi [1,*], Shivashankar Khanapur [1], Siddesh V. Hartimath [1], Boominathan Ramasamy [1], Peter Cheng [1], Hui-Xian Chin [2], Jun-Rong Tang [1], You-Yi Hwang [2] and Edward G. Robins [1,3]

1 Institute of Bioengineering and Bioimaging (IBB), Agency for Science, Technology and Research (A*STAR), 11 Biopolis Way, #01-02 Helios, 138667, Singapore
2 Singapore Immunology Network (SIgN), Agency for Science, Technology and Research (A*STAR), 8A Biomedical Grove, Immunos, 138648, Singapore
3 Clinical Imaging Research Centre (CIRC), Yong Loo Lin School of Medicine, National University of Singapore, 14 Medical Drive, #B1-01, 117599, Singapore
* Correspondence: julian_goggi@ibb.a-star.edu.sg; Tel.: +65-6824-7093

Citation: Goggi, J.L.; Khanapur, S.; Hartimath, S.V.; Ramasamy, B.; Cheng, P.; Chin, H.X.; Tang, J.R.; Ong S.T.; Hwang Y.Y.; et al.. *Biomedicines* **2022**, *14*, x. https://doi.org/10.3390/xxxxx
Academic Editor(s)::

Received:
Accepted:
Published: date

Publisher's Note: MDPI stays neutral with regard to jurisdictional claims in published maps and institutional affiliations.

Copyright: © 2022 by the authors. Submitted for possible open access publication under the terms and conditions of the Creative Commons Attribution (CC BY) license (https://creativecommons.org/licenses/by/4.0/).

Supplementary Materials

1.1 Flow Cytometry Markers

Kv1.3 (Kv1.3 potassium channel marker - polyclonal FITC; Sigma-Aldrich), CD103 (epithelial cell and DC subset marker - clone M290 FITC; BD Biosciences), CD25 (IL2 receptor marker - clone PC61 BB700; BD Biosciences), CD45 (pan-leukocyte marker - clone 30-F11 BUV395; BD Biosciences, New Hampshire, USA), Fixable Live/Dead Blue (Invitrogen, Singapore), CD62L (naïve T cell marker - clone MEL-14 BUV563; BD Biosciences), CD86 (macrophage marker - clone GL1 BUV615; BD Biosciences), F4/80 (pan-macrophage marker - clone T45-2342; BD Biosciences), NKp46 (pan-NK cell marker - clone 29A1.4 BUV737; BD Biosciences), CD3 (pan-T cell marker - clone 500A2 BUV805; BD Biosciences), FoxP3 (T regulatory cell marker - clone 150D AlexaFluor647; Biolegend, San Diego, CA, USA), CD44 (stem cell and T activation marker - clone IM7 APC-R700; BD Biosciences), CD11b (myeloid marker - clone M1/70 APC-Cy7; Biolegend), Granzyme B (Granzyme B marker - clone QA16A02 PE; Biolegend), CCR7 (memory T cell marker - clone 4B12 PE-CF594; BD Biosciences), CD19 (pan-B cell marker - clone 6D5 PE-Cy5; Biolegend), CD206 (macrophage marker - clone C068C2 PE-Cy7; Biolegend), CD127 (IL7 receptor alpha chain, memory and effector T cell marker - clone SB/199 BV421; BD Biosciences), Ly6G (neutrophil marker - clone 1A8 BV480; BD Biosciences), CD8 (cytotoxic T cell marker - clone 53-6.7 BV510; BD Biosciences), CD11c (dendritic cell marker - clone N418 BV570; Biolegend), Ly6C (macrophage marker - clone HK1.4 BV605; Biolegend), Siglec F (eosinophil marker - clone E50-2440 BV650, BD Biosciences), CD68 (macrophage marker - clone FA-11 BV711; Biolegend), CD4 (helper T cell marker - clone GK1.5 BV750; BD Biosciences), I-A/I-E (MHC class II marker - clone M5/114.15.2 BV785; Biolegend).

1.2 Dimension reduction analysis

t-Distributed Stochastic Neighbor Embedding (t-SNE) was used for unbiased dimension reduction and Rphenograph was used for clustering. t-SNE, clustering and overlay with t-SNE maps were performed with the cytofkit package in RStudio (3) (https://github.com/JinmiaoChenLab/cytofkit). The default cytofkit parameters was used for the analysis on 1000 cells from each fcs file. Rphenograph clustering was performed for the following markers: Kv1.3, CD3, CD4, CD8, CD11b, CD11c, CD206, F4/80, Granzyme B, I-A/I-E, Ly6C, Ly6G, Nkp46 and Siglec-F.

Tables

Treatment arm	Days post inoculation	Tumour volume (mm^3 ± SD)
**Control**	6	149.16 ± 17.29
	9	299.44 ± 59.94
	12	888.38 ± 283.37
	15	1228.32 ± 355.74
	18	1511.52 ± 403.46
	21	1803.16 ± 459.17
**Treatment Responders (TR)**	6	143.90 ± 23.09
**αPD1**	9	294.83 ± 44.63
	12	342.27 ± 150.07
	15	355.91 ± 185.23
	18	368.84 ± 228.10
	21	383.77 ± 261.91
**OXA**	6	149.00 ± 19.04
	9	193.43 ± 87.07
	12	340.67 ± 201.33
	15	354.82 ± 225.13
	18	369.07 ± 250.03
	21	392.26 ± 283.33
**αPD1 + OXA**	6	155.37 ± 24.55
	9	254.93 ± 15.90
	12	244.61 ± 84.58
	15	237.02 ± 150.24
	18	259.50 ± 183.87
	21	266.64 ± 181.08
**5-FU**	6	148.00 ± 18.03
	9	233.99 ± 47.05
	12	313.17 ± 83.97
	15	349.16 ± 127.94
	18	406.71 ± 209.68
	21	456.27 ± 266.19
**αPD1 + 5-FU**	6	149.03 ± 19.45
	9	184.36 ± 45.37
	12	201.36 ± 93.22
	15	212.93 ± 124.58
	18	243.62 ± 189.36
	21	245.01 ± 182.66
**Treatment Non Responders**	6	148.77 ± 13.28
**(TNR)**	9	319.74 ± 69.34

	12	648.90 ± 171.26
	15	858.82 ± 239.00
	18	1130.50 ± 296.91
	21	1344.91 ± 359.75

Supplementary Table S1. Summary of tumour volumes in controls, ICI treatment responders (TR) and treatment non-responders (TNR).

	**Treatment Responders (TR)/ Treatment Non-Responders (TNR) (No. mice)**
**ICI Treatment**	**CT26**
Control	0/10
αPD1	6/15
OXA	7/10
αPD1 + OXA	8/10
5-FU	6/10
αPD1 + 5-FU	7/10
% Overall Therapy Response	61.8%

Supplementary Table S2. Summary of ICI treatment responders (TR) and treatment non-responders (TNR) across all therapy arms in syngeneic CT26 and MC38 colon cancer models

**Treatment arm**	**%TGI (mean ± SD)**
αPD1	85.82 ± 15.83
OXA	85.30 ± 17.13
αPD1 + OXA	92.89 ± 18.80
5-FU	81.43 ± 16.09
αPD1 + 5-FU	94.20 ± 12.86
TNR	27.71 ± 21.75

Supplementary Table S3. Tumour growth inhibition % on day 21 for each treatment arm compared to control.

A

	**CD4⁺ % of CD3⁺**	**CD4⁺ Teff% of CD4⁺**	**CD4⁺ T$_{CM}$ % of CD4⁺**	**CD4⁺ Treg % of CD4⁺**
**Control**	39.33 ± 9.97	48.41 ± 17.47	9.40 ± 5.32	11.78 ± 3.96
**<u>TR</u>**				
**αPD1**	25.85 ± 4.37	20.86 ± 12.90	25.64 ± 2.26	23.76 ± 7.84 **
**OXA**	12.78 ± 4.39	17.95 ± 16.12	27.20 ± 9.54	29.24 ± 6.91 **
**αPD1 + OXA**	28.35 ± 10.20	24.91 ± 12.91	22.98 ± 7.23	24.63 ± 4.58 **
**5-FU**	64.94 ± 4.25	45.85 ± 13.01	8.54 ± 3.45	10.25 ± 4.27
**αPD1 + 5-FU**	62.32 ± 5.71	40.65 ± 12.09	14.41 ± 5.92	16.21± 1.88 *
**TNR**	41.89 ± 3.98	53.34 ± 10.28	6.20 ± 3.75	11.94 ± 1.12

B

	**CD8⁺ Teff% of CD8⁺**	**CD8⁺ T$_{CM}$ % of CD8⁺**	**F4/80⁺ % of CD45⁺**	**Eos % of CD45⁺**
**Control**	34.74 ± 6.80	1.01 ± 0.35	6.00 ± 1.43	1.07 ± 0.15
**<u>TR</u>**				
**αPD1**	52.86 ± 16.73 *	2.29 ± 1.09	3.94 ± 1.33	1.73 ± 0.30
**OXA**	66.32 ± 11.76 *	0.64 ± 0.43	2.34 ± 1.11 *	0.94 ± 0.34
**αPD1 + OXA**	58.86 ± 17.30 *	1.25 ± 0.77	3.31 ± 1.32 *	1.42 ± 0.51
**5-FU**	51.97 ± 10.37 *	1.97 ± 1.12	2.15 ± 1.01 *	2.14 ± 0.50 *
**αPD1 + 5-FU**	46.84 ± 9.22 *	2.18 ± 1.52	1.69 ± 1.05 *	2.54 ± 0.67 *
**TNR**	32.57 ± 7.60	1.06 ± 0.85	6.28 ± 1.12	0.98 ± 0.33

Supplementary Table S4. Table showing the tumour associated immune cell populations from CT26 tumour-bearing mice at day 12 post-induction of αPD1 monotherapy or combination therapies. **A**. Percentages of CD4⁺, CD4⁺ T-effector, CD4⁺ T-central memory and CD4⁺ T-regulatory immune cell subpopulations and **B**. Percentages of CD8⁺ T-effector, CD8⁺ T-central memory, F4/80⁺ and Eos⁺ immune cell subpopulations are shown across control groups, treatment responders (TR) and treatment non-responders (TNR) across all treatment arms. Data are shown as mean % of cells ± S.D. and are representative of n=5-10 mice/ group, * P<0.05; ** P<0.01 comparing TR to TNR.

**Treatment arm**	**Days post inoculation**	**Tumour volume (mm³ ± SD)**
**Reimplanted TNR**	22	120.45 ± 12.17
	27	207.51 ± 15.43
	30	269.28 ± 9.67
	34	384.46 ± 215.10
	37	593.31 ± 338.70 *
**Reimplanted TR**	22	112.64 ± 24.74
	27	195.65 ± 65.88

	30	176.49 ± 39.94
	34	229.97 ± 47.02
	37	235.49 ± 24.34

Supplementary Table S5. Summary of tumour volumes in reimplanted TNRs and reimplanted TRs. Data are displayed as mean ± S.D. (TR, treated responder; TNR, treated non-responder) and are representative of n=4-6 mice/ group, * $P<0.05$ comparing TR to TNR

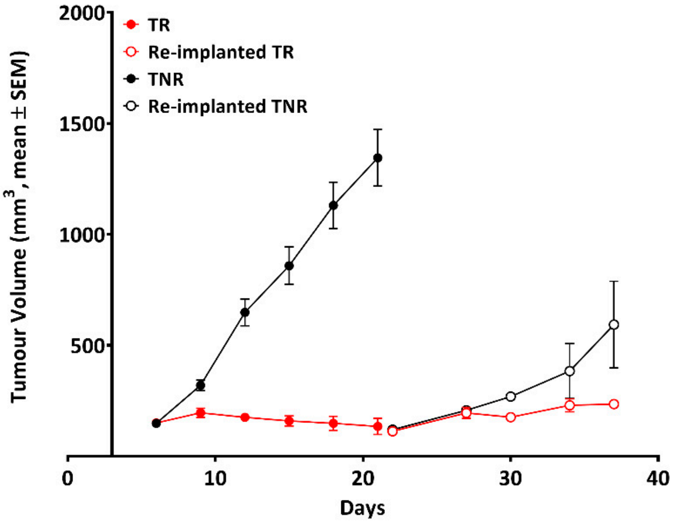

Supplementary Figure S1. Average tumour volumes showing initial tumour growth (TR, red closed circles and TNR, closed black circles) and after CT26 tumour cell re-implantation subcutaneously into the contralateral shoulder of TRs (red open circles; animals with high [^{18}F]AlF-NOTA-KCNA3P uptake >0.8%ID/g) or TNRs (black open circles; animals with low [^{18}F]AlF-NOTA-KCNA3P uptake <0.5%ID/g). Data are displayed as mean ± S.E.M. (TR, treated responder; TNR, treated non-responder).
